# Bat vocal sequences enhance contextual information independently of syllable order

**DOI:** 10.1016/j.isci.2023.106466

**Published:** 2023-03-21

**Authors:** Yoni Amit, Yossi Yovel

**Affiliations:** 1 School of Zoology, Faculty of Life Sciences, Tel Aviv University, Tel Aviv, Israel; 2 Sagol School of Neuroscience, Tel Aviv University, Tel Aviv, Israel; 3 National Research Center for Biodiversity Studies, The Steinhardt Museum of Natural History, Tel-Aviv University, Tel Aviv, Israel

**Keywords:** Biological sciences, Zoology, Evolutionary biology

## Abstract

Many animals, humans included, rely on acoustic vocalizations for communication. The complexity of non-human vocal communication has been under debate one of the main open questions being: What could be the function of multi-syllabic vocal sequences? We address these questions by analyzing fruit-bat vocal communication. We use neural networks to encode the vocalizations, and statistical models to examine the information conveyed by sequences of vocalizations. We show that fruit-bat vocal sequences potentially convey more contextual information than individual syllables, but that the order of the syllables within the sequence is unimportant for context. Specifically, sequences are composed of slightly modified syllables, thus increasing the probability of context-specificity. We note that future behavioral, e.g., playback experiments are needed in order to validate the biological relevance of our statistical results. We hypothesize that such sequences might have served as pre-syntax precursors in the evolution of animal communication.

## Highlights

• Fruit bats emit sequences of vocalizations while interacting with conspecifics
• Artificial neural networks can be used to encode bat vocalizations
• Longer sequences of vocalizations convey more information about their context
• The order of the syllables in the sequence does not seem to affect information

## Graphical abstract

**Figure:**
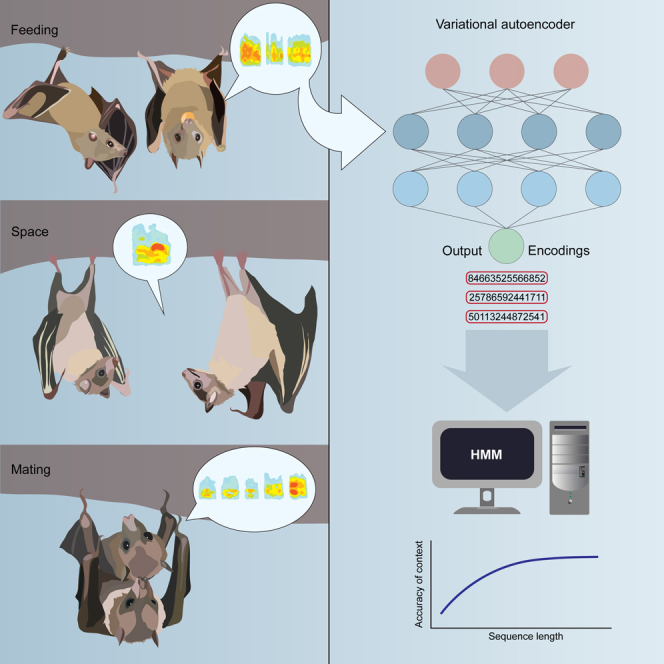

## Introduction

Animals often emit sequences of social vocalizations. The function of such vocal sequences and how they evolved from single vocalizations is currently unknown. Many previous studies have suggested that vocal sequences are not random; that is, they are not composed of a random set of syllables from the animal’s repertoire. The regularities defining non-random sequences are often referred to as the “syntax” of the animal communication system.^1^^,^^2^^,^^3^ In its widest definition, as adopted in this paper, animal communication syntax refers to any system of rules that orders a sequence of signals in a non-random manner.^1^^,^^2^^,^^3^^,^^4^ More complex communication systems include syntax that affects the meaning of the vocalizations; that is, communication systems in which syntax and semantics interact.^5^ Syntax is thus commonly graded according to its complexity. At the highest level is compositional syntax, which has only been shown for a handful of species,^5^^,^^6^^,^^7^^,^^8^ which combines meaningful units together into sequences that generate novel meaning.

Sequences and their regularities have been studied in birds^3^^,^^4^^,^^9^^,^^10^^,^^11^ and in many mammals including primates,^7^^,^^8^^,^^12^^,^^13^ cetaceans,^14^ hyraxes,^15^ mongoose,^16^ and bats.^17^^,^^18^^,^^19^ Many bats rely on vocalizations for intra-species social communication (e.g.,^20^^,^^21^^,^^22^) often emitting sequences of vocalizations. Several previous studies suggested that bat vocal sequences are not random. One such study showed that Mexican free-tailed bats emit sequences with different elements when they are directed at a passing bat vs. when they are uttered spontaneously.^23^ Another study focusing on the neural processing of vocal sequences in the bat auditory cortex, revealed that neurons respond when the animal is exposed to certain sequences of vocalizations but not to others.^17^ A third study examined the ontogeny of the production of bat vocal sequences, and found a human-like babbling phase in which sequences or vocalizations are uttered by newborn pups.^24^ However, none of these studies examined the potential information that might be conveyed by sequences of bat vocalizations, which was the goal of the present study.

**Figure 1:**
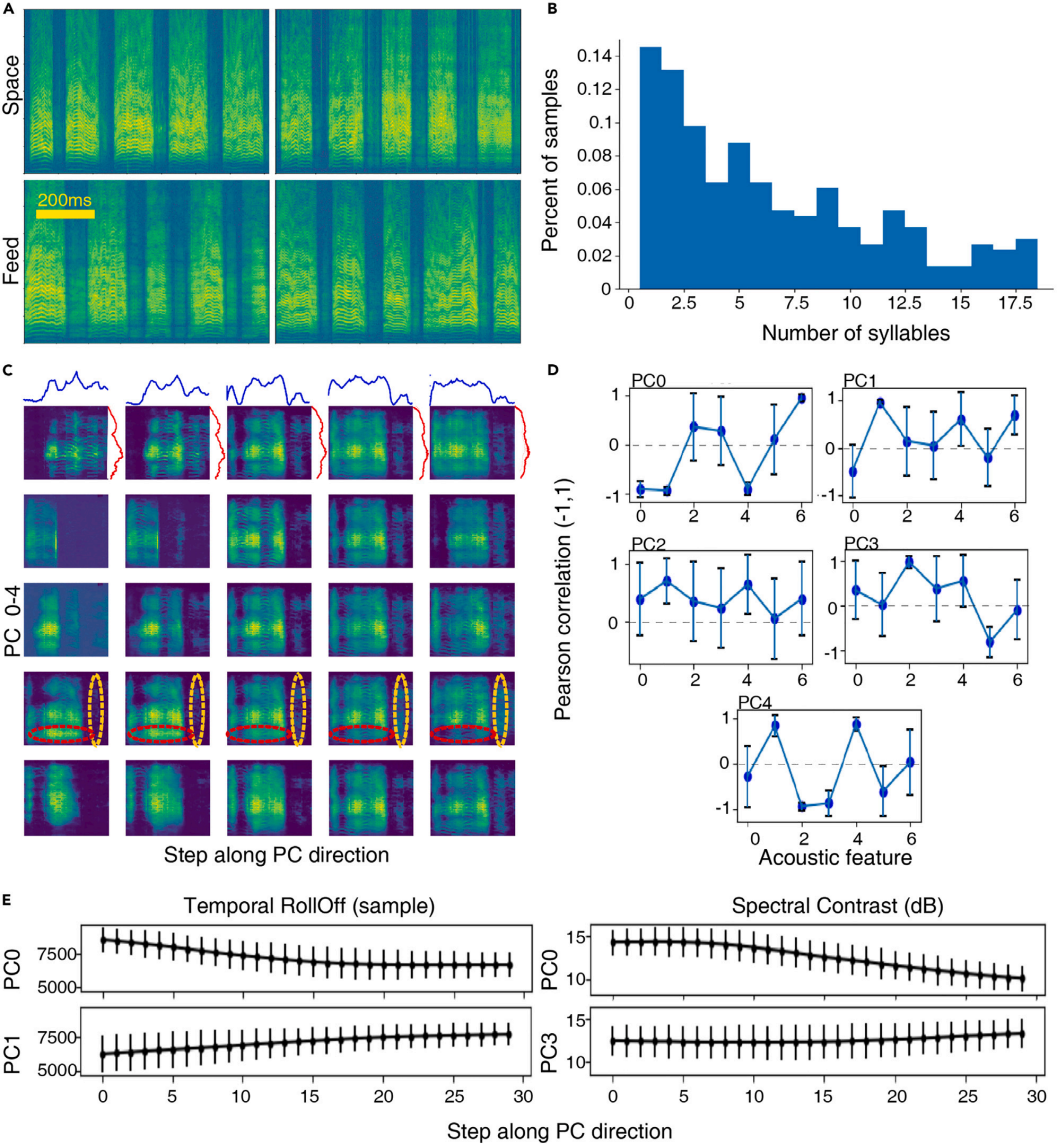
Acoustic representation of bat vocalizations using neural networks (A) Four representative sequences of fruit-bat vocalizations uttered in two contexts. See typical interactions in Videos S1, S2, and S3. (B) The distribution of the number of syllables in fruit-bat vocal sequences. (C) The effect of the first top five PCs on a random syllable is presented (PC weight increases from left to right). The blue and red lines above the first row of spectrograms depict the temporal and spectral envelopes (computed by projecting the spectrogram on the X or Y axes, respectively). These two envelopes are proxies of the temporal roll-off and the spectral contrast respectively, and it can be seen how moving along PC1 (from left to right) elongates the syllable and flattens the spectrum, thus reducing spectral contrast. The orange and red ellipses in the fourth row demonstrate the addition/removal of a temporal phoneme-like feature and a low frequency formant-like spectral feature, respectively. (D and E) (D) The correlation of the first five PCs with seven acoustic features (X axis, see STAR Methods) revealed that the temporal roll-off and the spectral contrast were most correlated—see examples in panel (E), where we varied the PC weight and examined the effect on these two acoustic features. Lines and bars represent means + STDs.

Focusing on the Egyptian fruit bat, we set out to determine the role of the sequence in bat vocal communication and to obtain new insight into its evolution. Egyptian fruit bats roost in large colonies that can be inhabited by thousands of individuals, which frequently emit sequences of vocalizations as part of their social interactions. Such sequences are composed of a series of up to ∼20 vocalizations (henceforth syllables) with (100–200 ms) intervals of silence between them (Figures 1A and 1B). Sequences are separated from each other by much longer (at least 1 s but often many minutes) intervals. The great majority of vocalizations in this species are uttered during agonistic interactions in the colony, where each sequence accompanies a single agonistic interaction, and yet, manifests different types of information (Videos S1, S2, and S3 which each demonstrate a single interaction in the contexts: feeding, mating, and space respectively). In a previous study carried out by our lab, Prat et al. showed that fruit-bat vocalizations contain information about the identity of the individual emitter, about the context in which they were uttered, and to some extent also about the outcome of the interaction.^25^ Specifically, it was shown that vocalizations uttered during agonistic interactions over food, space or mating can be distinguished. However, in that study, the acoustics of the vocalizations were analyzed in short time windows only, and thus, the importance of the sequence for conveying information and their statistical regularities were never examined. Because in the previous study we have already demonstrated that short vocal segments contain considerable information about the identity of the emitter, here, we focus on the contextual information conveyed by the sequences.

Video S1. An example of a feeding interaction including the accompanying vocalizations, related to Figure 1A

Video S2. An example of a mating interaction including the accompanying vocalizations, related to Figure 1A

Video S3. An example of an interaction regarding space including the accompanying vocalizations, related to Figure 1A

Detecting repeating elements (i.e., categorization of vocalizations) of an animal’s communication system is usually a prerequisite for studying syntax.^26^^,^^27^^,^^28^ One of the most common methods to achieve this is to manually scrutinize the recorded vocalizations and to group syllables based on their visual similarities. This method has been used in numerous studies on song-birds and other species, as well as in most of the previous bat studies.^23^^,^^29^ Unlike song-bird vocalizations, fruit-bat (and many other mammalians) vocalizations are non-tonal and have relatively low fundamental frequencies.^25^ They are thus characterized by numerous noisy harmonics. This makes them especially challenging for categorization, and thus ill-suited for visual identification of repeatable syllables (see examples in Figure 1A). Here, we used a combination of deep-learning algorithms and Hidden-Markov-Models (HMMs) in order to embed fruit-bat vocalizations in a lower-dimensional feature space and to examine the order of vocal sequences and their role in conveying information. We show that while grouping syllables into sequences improves context classification, the order of the syllables within the sequence, does not affect context classification. We suggest that such sequences of vocalizations might have appeared early on during the evolution of animal vocal communication. We note that our analysis is only statistical at this stage, and requires behavioral experiments for validation.

## Results

We adopted a non-supervised deep-learning algorithm to encode the syllables into a lower-dimensional feature space. Specifically, we used a conditional variational autoencoder (CVAE) to encode the syllables into a 512-dimensions vector. The values of this vector can be thought of as the equivalent of routinely used acoustic features (e.g., spectral peak). However, when using a neural network (such as a CVAE), the features usually represent complex spatiotemporal features. Notably, the CVAE was trained with spectrograms of single syllables while taking the emitter’s identity into account (as the condition). This procedure is common in human speech analysis^30^^,^^31^ and is crucial for representing inter-individual variability, which is often the main source of variability in such datasets. We analyzed recordings of three female adult fruit bats recorded continuously for 10 weeks generating a total of 28,847 syllables. This large dataset allowed us to capture much of the variance in the fruit-bat acoustic system.

The feature space produced by the CVAE can be thought of as a multi-dimension description of the acoustics of the fruit-bat communication system. To scrutinize this feature space, we ran a PCA (principal component analysis) analysis on the 512-dimensions and projected the encoded vectors onto the first 40 principal components (accounting for 42% of the variance). We then chose arbitrary vocal syllables and manipulated them by moving along each of these 40 PCs in order to examine the effect of each PC direction on the syllable (in Figure 1C, we present the effect of the five top PCs to exemplify their action). This analysis revealed that each PC encompasses multiple spectral and temporal acoustic features and cannot be explained by a single acoustic parameter. Furthermore, in order to determine acoustic information encoded by our embedding method, we manipulated random syllables by changing the weight of each PC in steps and measured the effect of this manipulation on seven temporal and spectral acoustic features (see STAR Methods). We found that many of the PCs were correlated with one or more of these seven acoustic features, demonstrating that the PCs encapsulate acoustic variance (Figures 1D and 1E).

The advantage of the CVAE representation in comparison to using specific acoustic features is that it allows capturing multi-feature acoustic variability. The two most correlated acoustic features were the temporal roll-off, which is related to the duration of the syllable, and the spectral contrast, which is related to the uniformity of the spectrum (the mean Pearson p-value over all 40 PCs was <0.001 for both of these acoustic features). Indeed, scrutinizing the effect of the first PC on a randomly chosen syllable (Figure 1C) reveals how this PC changes both the duration and the spectral contrast of the syllable (compare the blue and red lines above and on the side of the spectrograms, representing the duration and spectral uniformity respectively).

**Figure 2:**
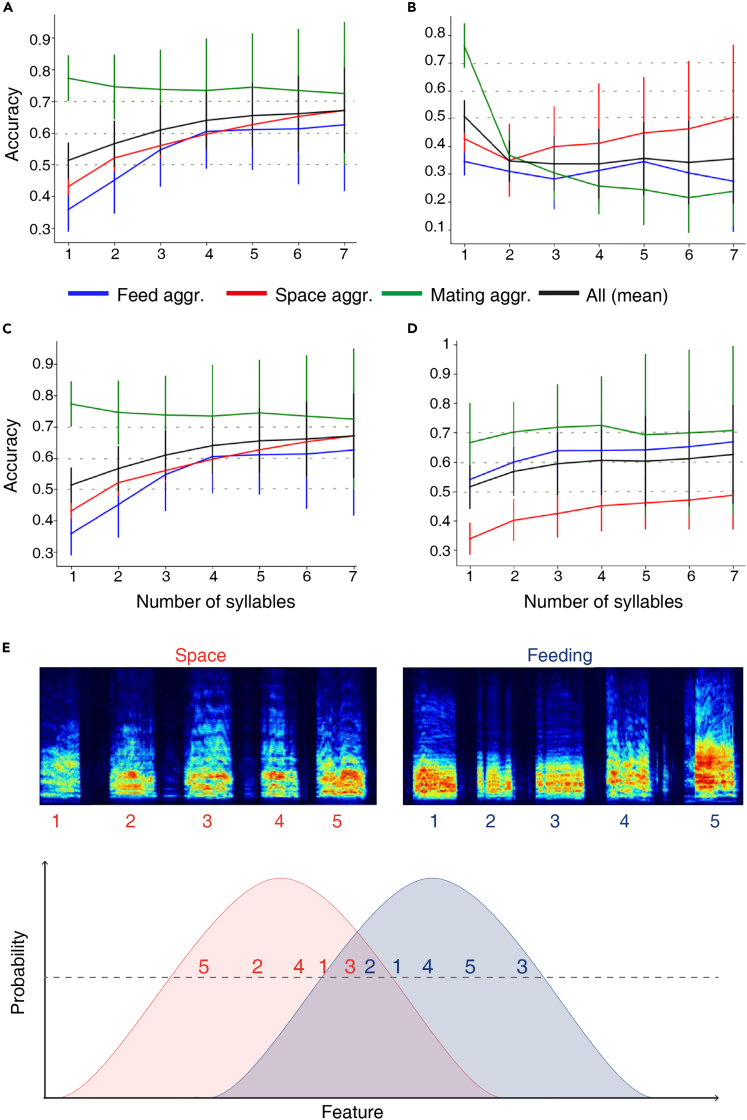
Sequences of information (A–D) HMM classification (on the test set only) as a function of the number of syllables (X axis) for three contexts (color-coded—see legend). Black line shows the balanced accuracy for all three. (A) Original data. (C) Permuted sequences where syllables are randomly moved between sequences but their position within the sequence remains the same. Note that the 1-grams were not permuted and thus provide the same information as in “A”. (C) Permuted sequences where the order of the syllables within the sequences was randomly shuffled. Results are identical to in “A”. (D) Sequences represented by seven acoustic features (instead of VAE’s). Lines and bars represent meanrs + STDs. (E) A schematic suggesting why sequences contribute to context conveyance. The red and blue shaded areas represent hypothetical distributions of several (hypothetical) features for two different behavioral contexts. The numbers represent the order of syllables taken from the two sequences shown above the distributions. Despite much overlap between the distributions, some syllables within the sequence (e.g., 3 blue and 5 red) will fall near the margins of the distribution making classification easier. The schematic depicts one feature, but the feature space is actually multi-dimensional.

In all of the following analyses, we thus used the 40-dimensional vectors (PC-weights) generated by this method to represent each syllable. Below, we also present all the analyses for a representation of the vocalizations that are based on a set of specific acoustic features (instead of the CVAE). Next, we sought to determine whether sequences of vocalizations convey more contextual information than single syllables. We used annotated sequences of vocalization that were uttered by the bats in one of the three contexts (most commonly observed in our colony): fighting over food—when an individual attempts to scrounge from another individual; over space—when a bat enters the individual space of another bat; or before mating, when a female responds aggressively to mating attempts. We will refer to these three contexts as feeding, space, and mating respectively. We trained a multivariate-Gaussian-HMM model with three hidden states representing the three contexts noted above (note that this HMM was trained using a supervised approach, see STAR Methods). We trained the HMM model with 326 sequences comprising a total of 2,953 syllables. We divided each sequence into all possible n-grams (yielding a total of 12,900 n-grams). We then tested the HMM’s context classification on sequences with increasing length (between 1 and 7 syllable n-grams). The HMM model was able to identify the context in which the vocalizations were uttered far above chance level (Figure 2A, the balanced accuracy (BA) for sequences of seven syllables was 66 ± 9% vs. 33% by chance, specifically 63 ± 17, 68 ± 16, 69 ± 19% for the feeding, space, and mating contexts). These results show mean ± SD for an 8-fold cross-validation procedure in which 87.5% of sequences are used for training and the rest for testing each time. Notably, context classification improves when the sequences contain more syllables (overall and at least in two contexts—feeding and space). That is, the longer the sequence, the more information it conveys about the context (p = 1.2∗10^−10^, generalized linear model (GLM) with the accuracy set as the explained variable, the number of syllables, and the context set as fixed factors, and the cross-validation iteration as a random effect, see Tables S1 and S2). The differences between contexts were also significant, with feeding interactions recognized significantly less than the other two. We controlled for the effect of dividing the sequences into n-grams by training an HMM without this division (i.e., on the original sequences only). When doing so using an 8-fold cross-validation we obtained a similar performance, 61 ± 10, 63 ± 19, 83 ± 14% for the feeding, space, and mating contexts and an overall BA of 66 ± 10%. We also tested the overall performance for each individual separately (after training the HMM model on all data together), which revealed a similar average performance for the three individuals—55, 70, and 71% (in comparison to a chance level of 33%).

We then performed another control, in which we switched syllables between all sequences (across contexts) keeping their position in the sequence (e.g., we permuted all of the position 2 syllables between the sequences but always kept them in position 2, without changing any other parts of the training-testing procedure). In this case, longer sequences did not provide more contextual information validating the hypothesis that a random assembly of syllables would not convey contextual information (Figure 2B, average accuracy was at chance level, p = 0.63, GLM with the same variables as above).

We next examined whether the order of the syllables within a sequence contributes to context classification. To this end, we permuted the internal order of syllables within sequences and we then trained the same supervised context-HMM classifier (as aforementioned) with 8-fold cross-validation. This internal permutation did not affect the context classification performance of the HMM, suggesting that syllable order does not contribute to conveying contextual information. Context classification results, in this case, were identical to those of the original data with an accuracy of 63 ± 17, 68 ± 16, and 69 ± 19 for the feeding, space, and mating contexts and an overall BA of 66 ± 10 (Figure 2C).

To determine whether the model we trained can represent a form of compositional syntax, in which syllables with certain meanings (i.e., context) are combined into sequences to generate new meanings, we tested the (aforementioned) HMM model on each of the syllables within the sequences separately (i.e., on 1-grams) and compared their classified context to the context of the entire sequence. We found that the classified syllable context was the same as the context of the entire sequence negating compositional syntax. Specifically, more than 80% of the individual syllables were classified as belonging to the same context as the entire sequence. Thus, we conclude that, from a statistical point of view, individual syllables convey the same contextual information as the sequence, but because they are not identical acoustically, the sequence conveys more contextual information than a single syllable alone (see additional discussion in the following).

To determine whether the “simple” acoustic features that we extracted can also provide contextual information, we ran the same context-HMM model on these features (instead of the VAE embedding), either using each feature separately or using all seven features together. This analysis revealed that even a low dimensional acoustic representation of the syllables already provides contextual information and that using all seven features together provides similar contextual information to that when using the VAE embedding (the overall BA was 64 ± 10% vs. 66 ± 6% for the seven acoustic vs. the CVAE features, Figure 2D). Note that space vocalizations did not classify well when using acoustic features (<50%) suggesting that the CVAE represents the different contexts better on average. Note also, that sequences conveyed more contextual information than individual syllables also when using an acoustic feature-based representation (p < 6∗10^−6^, GLM as above, see Tables S1 and S2).

## Discussion

We found that vocal sequences uttered by fruit bats convey more contextual information than single vocalizations. This suggests that the syllables used in each context arise from a different (multi-modal) acoustic distribution. Notably, there is much overlap between the distributions of the features of syllables of different contexts (whether we used the CVAE or the simple acoustic features). Indeed, when plotting any of the features that we tested, they were always part of a continuous distribution rather than distributed in clusters. Fruit-bat vocalizations thus do not seem to form separate “words” (although it is also possible that we are not describing them in the relevant feature space of the bat). We thus suggest that longer sequences convey more contextual information because uttering more vocalizations increases the chances of producing a distinct context-specific syllable (i.e., from the non-overlapping margins of the distribution of the two contexts, see schematic in Figure 2E). Note that, when using an HMM-like model to classify context, concatenating multiple identical syllables would not convey more information about context. Because we found that the order of syllables within a sequence can be randomized without affecting context classification, we do not refer to fruit-bat sequences as characterized by syntax. While our results also refute the hypothesis that fruit-bat sequences could be considered a form of compositional syntax, we do not suggest that bats or even fruit bats cannot use compositional syntax, as might be revealed by future studies applying different feature space or different statistics.^7^ We thus describe a system in which animals combine elements (i.e., syllables) that are already informative on their own to form sequences that convey the same context as the individual syllables, but that combining them improves the transmission of information (more than repeating them). We note that it is likely that sequences also provide other information, which we did not test here, such as, regarding the arousal level or motivation of the emitting animal.

**Figure 3:**
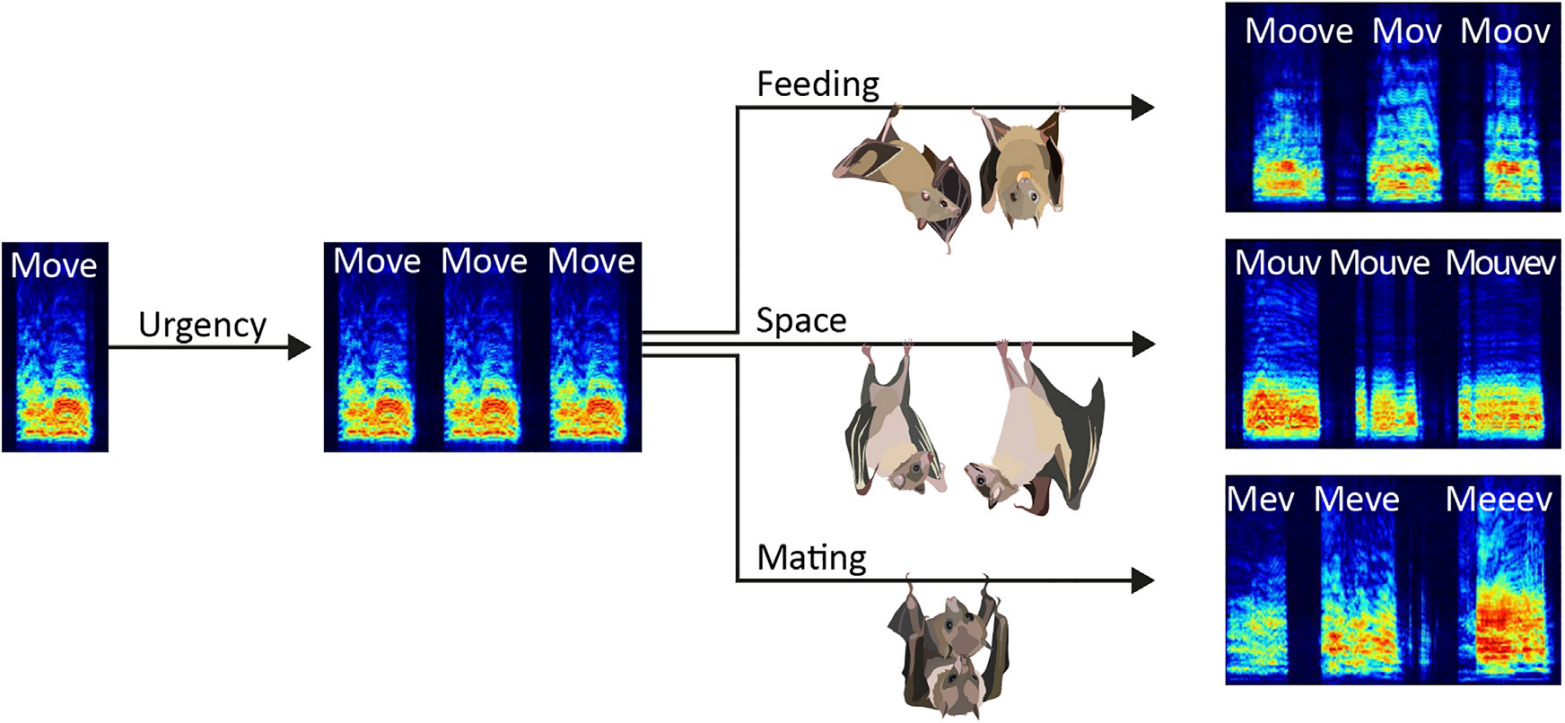
A conceptual framework for the evolution of animal vocal sequences We hypothesize that single vocalizations (“Move”) first evolved into sequences of identical vocalizations, and then modified into sequences of slightly different context-specific syllables.

In the next paragraph, we offer a speculative hypothesis regarding the evolution of such sequences. We hypothesize that this form of vocal sequences might be common in animals and might be a precursor to the evolution of syntax in animal communication (Figure 3). Let us imagine the ancestral fruit-bat colony in which the most common social interaction includes fighting over position in the cluster, and the vocal repertoire comprises only a single syllable, which we will refer to as “Move”. One could imagine that at higher arousal levels, an excited bat would repeat this syllable several times, uttering a sequence such as: Move-Move-Move. Such repeated signaling due to urgency is familiar to any pet holder and has also been documented in non-vocal communication, for instance, in orangutans.^32^ In the next phase, the n-repetition of the syllable might slightly change depending on the context of the interaction. For instance, when fighting over food the sequence might become Move-Mov-Mov and later perhaps Meve-Mov-Mev. This could be a result of the arousal level in this specific context (e.g., fighting while mating is more vigorous than fighting over place) or it could be a result of a physiological constraint, e.g., holding fruit in the mouth or calling while flying necessitates shortening the syllables. Over time, a sequence structure similar to the one we describe above might evolve in which a single syllable conveys contextual information, while a sequence of syllables conveys more information about the same context, because of the higher chance that one such syllable will be context-distinct. Eventually, a communication system will evolve in which the syllables in the sequence slightly differ from one another and the syllables in sequences of different contexts derive from different but overlapping distributions. This is somewhat reminiscent of a process termed “affixation” shown in primates, in which alarm syllables are modified (e.g., elongated) based on motivation and context, leading to a change in their meaning.^13^ Notably, several species of bats including Egyptian fruit bats have been shown to be vocal learners, i.e., they can modify their vocalizations based on exposure to sounds produced by others. Although vocal learning has mostly been studied in the context of individual syllables, it could also assist the establishment of certain sequences as well as the introduction of new variability into sequences.

Note that our case differs from what is sometimes referred to as “graded syntax” where the combination of syllables signals the degree of agitation in a specific context,^6^ because in our case, sequences convey different contexts (and not a single one). A system such as we describe here might be a precursor for evolving ordered sequences—or syntax—in which syllables within a sequence are not ordered randomly, as seems to be the case in fruit bats. However, much more comparative research is needed in order to support these ideas.

An alternative hypothesis regarding the evolution of sequences with syntax is the lexical constraint hypothesis,^8^^,^^33^ suggesting that when a species continuously increases the number of different syllables it utters, it will reach a point where further additions become uneconomical compared to combining already existing syllables, either due to production limits or memory limits. We find this hypothesis appealing from a theoretical point of view but also suggest that it ignores the fact that animal communication systems probably evolve from a single or a few syllables,^34^ which are thus likely to become first concatenated into sequences (of identical syllables), and only later modified to convey information. Many simple extant animal communication systems, such as dog barking, are mostly based on a single syllable that is modified occasionally based on arousal and other conditions. It is course also possible that different species have taken different evolutionary routes.

Encoding the acoustic properties of fruit-bat vocalizations using a neural network autoencoder to represent the syllables has revealed new insight into the complexity of fruit-bat communication. Acoustically, we show that both formant-like features and phoneme-like features exist in fruit-bat vocalizations. This is revealed for instance in PC 3, which seems to both add and remove low-frequency formant-like structures (see red ellipses in Figure 1C) and also to add and remove temporal phoneme-like features (see orange ellipses in Figure 1C).

Both syntax and semantics were traditionally thought to be unique to human language, but have since been shown to exist to some degree in other animal species.^5^ It has been suggested that compositional syntax evolved when callers and receivers share an interest in exchanging information.^6^ We accept this hypothesis, and suggest how the use of sequences could have evolved even in a social structure in which individuals typically do not operate as a group,^35^^,^^36^ but only roost together in aggregations. We have uncovered a simple form of sequences that conveys contextual information in fruit bats, despite the lack of clearly distinguishable syllables and order within the sequence. Our statistical analysis should be followed by behavioral experiments in order to validate our findings. This study, however, has touched upon one of the fundamental questions in animal communication, namely, what is the basic unit of information while demonstrating a system in which a sequence of multiple units exemplifies the information that is already conveyed by a single syllable. Such sequences might have served as precursors for sequences with more developed regularities.

### Limitations of the study

One major limitation of this study is that the features extracted by the VAE neural network that we used to encode bat vocalizations might not be the optimal ones. The bat’s brain has probably evolved over a long time period to extract information from social vocalizations. Similar to our VAE, the brain is a non-linear machine, but the encoding that it uses might be completely different from ours and probably extracts much more information. A second and related limitation of this study is the lack of behavioral evidence to support our statistical findings. Behavioral validation is essential in order to prove that our findings are relevant for the animals.

## STAR★Methods

### Key resources table

**Table:** 

REAGENT or RESOURCE	SOURCE	IDENTIFIER
**Deposited data**		

Original wav files	Previous study	Prat et al.^37^, Scientific data

Acoustic syllable encodings	Self-recordings	https://data.mendeley.com/datasets/mjfv43zgtv/3

**Experimental models: Organisms/strains**		

Three female Egyptian fruit bats (*Rousettus aegyptiacus*)	Caught in a cave in central Israel	Taxonomy ID: 9407

**Other**		

Microphones + AD converters	Avisoft Bio-acoustics	CM16, SM1612

**Software and algorithms**		

Stats (GLMs) were run in Matlab 2019	The Mathworks	https://www.mathworks.com/downloads/;
All samples were randomized to control for possible biases. Exclusion was based on signal quality. The exact criteria are explained in the STAR Methods		

Self-written code	Self-written code in Python	https://data.mendeley.com/datasets/mjfv43zgtv/3

### Data

The data include recordings of 3,601 communication sequences (accounting for a total of 28,847 syllables) recorded from 3 female adult bats in a previous study.^25^ All raw annotated recordings (wav files) can be found here.^37^ The original recording were performied in insulated anechoic chambers in small groups of <10 bats in order to assure high quality recordings with little background noise. The pre-processing of the recordings included selecting sequences where the emitter and context are clear and without loud background noise (see^25^). We used the segmentation into syllables provided in the original paper. Each syllable was then transformed into an amplitude spectrogram using the STFT function (with a window length of 0.007 sec). Spectrograms were trimmed or zero-padded if necessary to create 256 × 640 images (representing 0.5 second segments with a frequency resolution of ∼140 Hz). These were used as the input for a Conditional Variational Autoencoder neural-network (CVAE, see next paragraph). All analyses were performed with Python. Neural network analyses were done using Python Keras^38^ and HMMs were fit using the Pomegranate and HMMlearn Python packages.

### Encoding

The CVAE neural network was composed of seven convolutional layers (in the encoder) and another eight in the decoder (see key resources table for a link to the full code). We only used high Signal-to-Noise-Ratio syllables to train the CVAE. To this end, we added a 0.05V threshold relative to the noise in order to remove weak syllables. This additional processing removed 57% of the syllables. This procedure was only relevant for the training of the CVAE, while (unless stated otherwise) all analyses were performed on all syllables. The CVAE beta parameter was gradually increased following the KL-annealing procedure from 0.1 to 1 (see https://arxiv.org/abs/1903.10145). A CVAE network learns a probabilistic mapping between a syllable represented by a (256∗640) spectrogram and a latent 512 feature space vector (referred to as the embedding) while accounting for the emitter of each vocalization (the Condition). We used 80% of the spectrograms for training and 20% of them for testing the network.

### PCA

We used a PCA analysis in order to reduce the 512 feature space to a 40 dimensional space that accounted for 42% of the variance. In order to explain the variance encapsulated by our PC’s, we chose random real syllables and moved along each of the first five leading-PC directions to illustrate their effect. We used the CVAE autoencoder to decode the equivalent 512 embedding-vectors back to spectrograms. Specifically, the autoencoder enables converting encoding vectors to syllables and vice versa. Thus, given a 40-dimension vector, we can convert it to a 512-dimension encoding using the PCs and then convert it into a syllable using the autoencoder.

### Comparison with acoustic features

In order to estimate the effect of these leading PCs on the acoustics of the vocalizations, we estimated the correlation between changing the PC and the seven following acoustic features (each of them estimated for the entire manipulated syllable). Unlike the vocalization systems of some animals (e.g., song-birds, mice and some insectivorous bats), fruit-bat vocalizations are what we usually term ‘noisy’ and thus their fundamental frequency (or pitch) is not easy to estimate. For the same reason, it is difficult to talk about frequency modulation.

1) Spectral contrast^39^ – the difference between the mean energy in the top quantile (peak energy) of the spectrum to that of the bottom quantile (valley energy).
2) Temporal centroid^40^ defined as:

$$Centroid=\frac{\sum_{n=0}^{N-1}t\left(n\right)x\left(n\right)}{\sum_{n=0}^{N-1}x\left(n\right)}$$

where*x(n)* represents the magnitude of bin *n*, and *t(n)*represents the time of that bin

3) Spectral centroid^40^ defined as:

$$Centroid=\frac{\sum_{n=0}^{N-1}f\left(n\right)x\left(n\right)}{\sum_{n=0}^{N-1}x\left(n\right)}$$

where*x(n)* represents the magnitude of bin *n*, and *f(n)*represents the center frequency of that bin.

4) The spectral rolloff^41^ is defined as the center frequency of a spectrogram bin such that at least 0.85 of the energy of the spectrum in this frame is contained in this bin and the lower frequencies.
5) The temporal rolloff is defined as the center time of a time bin such that at least 0.85 of the temporal energy in this frame is contained in this bin and the in earlier times. This feature is a good approximation of the duration of the syllable.
6) The spectral bandwidth^40^ is defined as:

$${\left(\sum_{k}S\left(k\right){\left(f\left(k\right)-f_{c}\right)}^{P}\right)}^{\frac{1}{P}}$$

where S(k) is the spectral magnitude at frequency bin k, f(k) is the frequency at bin k, and fc is the spectral centroid. We used p = 2, and thus this is equivalent to a weighted standard deviation.

7) The Spectral flatness,^42^ also known as Wiener entropy, which quantifies how tone-like a sound is, as opposed to how noise-like.

To determine which acoustic features contribute most to the variance, we computed the Pearson correlation of each PC and the above acoustic features; that is, for 100 syllables, we varied the syllables by moving along each PC and computed the respective value of the acoustic feature. We then selected the features with the lowest Pearson p-values.

### Examining context using HMMs

Using the trained CVAE, we encoded the syllables (without filtering weak syllables) into sequences of N∗512 (where N is the number of syllables in the acoustic sequence). Each sequence of syllables was then translated into a sequence of PC-weights (where each syllable is encoded by 40 PC weights). Here, we only used sequences annotated for three contexts – feeding aggression, general fighting and mating aggression, as provided in ref. 37 comprising a total of 326 sequences. We extracted all (n = 1–7) n-grams from the sentences using a sliding window (resulting in a total of 12,900 n-grams, but we also controlled for this step by running the entire procedure on the original data only). We trained a 3-hidden state multivariate Gaussian HMM, using a supervised approach. That is, we trained the HMM such that each hidden state is equivalent to one of the three annotated contexts (*feeding, fighting* or *mating*). We evaluated the accuracy of this model on the test set and estimated the performance for every n-gram separately. We performed an 8-fold cross-validation procedure, each time randomly selecting 87.5% of the data for training.

To examine the **compositional syntax** hypothesis we ran the above-noted trained context-HMMs on each syllable in the sequences separately. We then examined (using a binomial test) whether the probability of a syllable being classified as belonging to a context of the respective sequence was higher than expected by chance (0.33). For example, we tested whether the syllables in mating sequences were also classified as mating syllables above chance.

### Statistics

To test the effect of the number of syllables in a sequence on context recognition accuracy, we used generalized linear models (GLMs) with the accuracy of classification set as the explained variable and the number of syllables, the context and their interaction set as fixed factors. We used a logistic link function because the explained variable is a proportion. This analysis was also used for the different permutation controls.
