# Supplementary material for: Bat vocal sequences enhance contextual information independently of syllable order
Source: iScience. 2023 Mar 21;26(4):106466. doi: 10.1016/j.isci.2023.106466 (PMC10139886; doi:10.1016/j.isci.2023.106466)
Supplement: Document S1. Tables S1 and S2 [file mmc1.pdf]

iScience, Volume *26*

Supplemental information

Bat vocal sequences enhance contextual information independently of syllable order

Yoni Amit and Yossi Yovel

Supplementary table 1: **GLM results for the VAE sequences, related to Figure 2**

Distribution Normal

Link Logit

FitMethod MPL

Formula: accuracy ~ 1 + gram + context_cat + (1 | Validation)

Model fit statistics:

AIC	BIC	LogLikelihood	Deviance
366.08	389.96	-176.04	352.08

Fixed effects coefficients (95% CIs):

Name	Estimate	SE	tStat	DF	pValue	Lower	Upper
Intercept	-0.46034	0.093245	-4.9368	219	1.5728e-06	-0.64411	-0.27656
gram	0.1137	0.016819	6.7603	219	1.2315e-10	0.080556	0.14685
context_Space	0.34612	0.087474	3.9568	219	0.00010264	0.17372	0.51851
context_cat _Mate	1.2672	0.10649	11.899	219	1.6436e-25	1.0573	1.4771
context_cat_All	0.39059	0.087822	4.4475	219	1.3815e-05	0.2175	0.56367

Random effects covariance parameters:

Group: Validation (8 Levels)

Name1	Name2	Type	Estimate
{'(Intercept)'}	{'(Intercept)'}	{'std'}	0.059578

Group: Error

Name	Estimate
{'sqrt(Dispersion)'}	0.11262

Supplementary table 2: **GLM results for the Acoustic-feature analysis, related to Figure 2**

Distribution Normal

Link Logit

FitMethod MPL

Formula: accuracy ~ 1 + gram + context_cat + (1 | Validation)

Model fit statistics:

AIC	BIC	LogLikelihood	Deviance
366.08	389.96	-176.04	352.08

Fixed effects coefficients (95% CIs):

Name	Estimate	SE	tStat	DF	pValue	Lower	Upper
Intercept	0.34161	0.15925	2.1452	219	0.033039	0.027762	0.65547
gram	0.077741	0.016758	4.639	219	6.023e-06	0.044713	0.11077
context_Space	-0.98132	0.092461	-10.613	219	1.6874e-21	-1.1635	-0.79909
context_Mating	0.27735	0.1002	2.7679	219	0.006125	0.079864	0.47483
context_All	-0.20911	0.093175	-2.2442	219	0.025819	-0.39274	-0.02547

Random effects covariance parameters:

Group: Validation (8 Levels)

Name1	Name2	Type	Estimate
{'(Intercept)'}	{'(Intercept)'}	{'std'}	0.36554

Group: Error

Name	Estimate
{'sqrt(Dispersion)'}	0.11076
